# Facile Preparation of β-Cyclodextrin-Modified Polysulfone Membrane for Low-Density Lipoprotein Adsorption via Dopamine Self-Assembly and Schiff Base Reaction

**DOI:** 10.3390/ma17050988

**Published:** 2024-02-21

**Authors:** Fei Fang, Haiyang Zhao, Rui Wang, Qi Chen, Qiongyan Wang, Qinghua Zhang

**Affiliations:** 1 College of Chemical and Biological Engineering, Zhejiang University, Hangzhou 310027, China; 3090102659@zju.edu.cn; 2 Research and Development Center, Zhejiang Sucon Silicone Co., Ltd., Shaoxing 312088, China; hycsea@163.com (H.Z.); 13989503732@163.com (R.W.); 13989552135@163.com (Q.C.)

**Keywords:** dopamine, cyclodextrin-modified, low-density lipoprotein

## Abstract

A facile method for the immobilization of β-cyclodextrin on polysulfone membranes with the aim of selectively adsorbing low-density lipoprotein (LDL) was established, which is based on the self-assembly of dopamine on the membrane followed by the Schiff base reaction with mono-(6-ethanediamine-6-deoxy)-β-cyclodextrin. The surface modification processes were validated using X-ray photoelectron spectroscopy and attenuated total reflectance Fourier-transform infrared spectroscopy. Surface wettability and surface charge of the membranes were investigated through the water contact angle and zeta potential analysis. The cyclodextrin-modified polysulfone membrane (PSF-CD) showed good resistance to protein solutions, as shown by the measurement of BSA adsorption. The assessment of BSA adsorption revealed that the cyclodextrin-modified polysulfone membrane (PSF-CD) exhibited excellent resistance to protein solutions. To investigate the adsorption and desorption behaviors of the membranes in single-protein or binary-protein solutions, an enzyme-linked immunosorbent assay was employed. The results revealed that the PSF-CD possessed remarkable adsorption capacity and higher affinity for LDL in both single-protein and binary-protein solutions, rendering it a suitable material for LDL apheresis.

## 1. Introduction

Low-density lipoprotein (LDL) serves as the primary carrier for cholesterol and fat within the circulatory system of humans. It is widely accepted that the LDL particle consists of two primary compartments: a hydrophobic core, constituted by triglycerides, cholesteryl esters and free unesterified cholesterol, and an amphipathic shell consisting of phospholipid, free unesterified cholesterol and a single copy of apolipoprotein B-100 [1,2,3]. Persistently heightened LDL levels have consistently emerged as a prominent risk factor in the advancement of atherosclerosis, while reducing LDL levels effectively lowers the morbidity [4,5]. Hence, the pursuit of efficacious and precise methods for reducing LDL levels and maintaining them within the optimal range has garnered global prominence over the years. At present, lifestyle modifications [6,7,8] and pharmacological interventions (e.g., statins [9], inclisiran [10], bempedoic acid [11]) constitute the predominant clinical approaches for LDL level management. Nonetheless, protracted pharmacotherapy often precipitates adverse effects, culminating in issues like acute coronary syndrome and muscular debility [12,13]. Furthermore, these treatments are ineffectual for patients who are medication-resistant or those afflicted by severe hyperlipidemia or familial hypercholesterolemia [14,15].

In situations where conventional approaches fall short, LDL apheresis is highly recommendable owing to its remarkable efficacy in lowering LDL cholesterol and achieving therapeutic objectives. Presently, six prevalent LDL apheresis technologies are accessible, with four relying on the adsorption principle—dextran sulfate adsorption, dextran sulfate direct perfusion, polyacrylate-coated polyacrylamide direct perfusion and immune-adsorption [16,17,18]—which underscores the pivotal role of the adsorption material as a determining factor in the therapeutic efficacy of LDL-apheresis systems. Consequently, considerable focus has been directed towards exploring cost-effective and efficient adsorbents, resulting in the emergence of diverse LDL ligands, including antibodies [19,20], heparin [21,22,23,24], chondroitin sulfate [25,26], amino acid [27], phospholipids [28,29], chitosan derivatives [30,31] and others. Typically, a predominant feature of low-density lipoprotein (LDL) adsorption materials involves the incorporation of anionic characteristics. This design is strategically oriented towards the selective binding to positively charged domains of apolipoprotein B-100 within LDL, facilitated by electrostatic interactions. Li et al. highlighted the existence of a LDL–saccharide interaction involving the LDL and the saccharide component [32]. Subsequently, an anionic glycosylated membrane was further fabricated by Fang et al., the surface LDL adsorption capacity of which could be modulated by manipulating the proportion of anionic sugar groups [33].

Cyclodextrins (CDs) are a type of polysaccharides derived from the enzymatic degradation of starch, featuring bucket-like structures that encompass hydrophilic outer surfaces and relatively hydrophobic cavities. They are economical, readily accessible, biocompatible, and biodegradable [34,35,36,37,38]. The elegant structure of cyclodextrins enables them to spontaneously form inclusion complexes with a variety of biological molecules, such as cholesterol and phospholipids, through multiple interactions [39,40,41,42]. Furthermore, the abundance of hydroxyl groups on the outer edge of CDs can serve as hydrogen bonding sites for hydrophilic polar groups such as amino groups and hydroxyl groups [36,43]. Given its distinctive structural characteristics, cyclodextrin is expected to exhibit a strong affinity for LDL. Attracted by the excellent properties of cyclodextrin, in our previous work, a β-cyclodextrin-modified Au surface was prepared via the self-assembled monolayer method to investigate the interaction between β-cyclodextrin and LDL [44]. Subsequently, an β-Cyclodextrin-modified electrochemical biosensor for LDL was successfully fabricated [45].

**Scheme 1:**
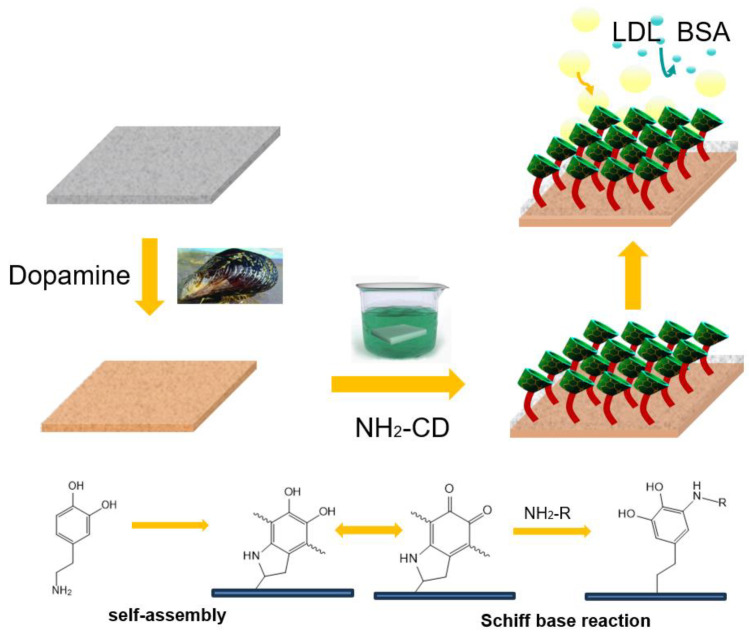
Schematic presentation for the preparation of β-cyclodextrin-modified polysulfone membrane for low-density lipoprotein adsorption via dopamine self-assembly and Schiff base reaction.

Polysulfone (PSF) membranes have found extensive utility in the field of hemodialysis [46]. Our research team is dedicated to the utilization of PSF membranes for enhancing hemodialysis with concurrent LDL removal, as well as developing biocompatible, economical, and effective adsorbents for LDL removal while simplifying the preparation methods to make them accessible for practical applications [21,22,23,47]. Herein, a β-cyclodextrin-modified polysulfone membrane for low-density lipoprotein adsorption was fabricated (see Scheme 1). First, by leveraging dopamine’s self-assembly properties, a polydopamine layer was generated on the surface of PSF membrane under mildly alkaline conditions. Given the abundance of benzoquinone groups within polydopamine, which provides a chemically versatile platform for establishing covalent bonds with -NH_2_ through Schiff base reactions, mono-(6-ethanediamine-6-deoxy)-β-cyclodextrin (NH_2_-β-CD) was used to induce the formation of a cyclodextrin functional layer via a Schiff base reaction. Surface properties of blank and modified PSF membranes were comprehensively examined using a combination of analytical techniques, including attenuated total reflectance Fourier transform infrared spectroscopy (ATR-FTIR), X-ray photoelectron spectroscopy (XPS), Zeta potential, water contact angle (WCA) and bovine serum albumin (BSA) adsorption experiment. An Enzyme-Linked Immunosorbent Assay (ELISA) was conducted to evaluate the performance of LDL adsorption and desorption on the membranes, as well as to investigate the interaction between the membrane surface and LDL.

## 2. Materials and Methods

### 2.1. Materials

LDL was procured from Millipore (Burlington, MA, USA). Dopamine (DA) hydrochloride, primary antibody anti-β-lipoprotein, human serum albumin (HSA) and secondary antibody anti-chicken (IgG) were obtained from Sigma-Aldrich. Mono-(6-ethanediamine-6-deoxy)-β-Cyclodextrin (NH_2_-β-CD) was acquired from Zhiyuan Biotechnology Co., Ltd. (Shandong, China). Polysulfone granules were provided by Zhejiang Qinyuan Water Treatment S.T. Co., Ltd. (Shanghai, China). Tris(hydroxymethyl)aminomethane was purchased from Amresco (Solon, OH, USA). Other chemicals were sourced from Sinopharm Chemical (Shanghai, China). Ultrapure water (18.2 MΩ cm) used in this study was prepared using an ELGA Classic UF system.

### 2.2. Preparation of β-Cyclodextrin-Modified PSf Membranes

PSF granules were dissolved in N-methyl-2-pyrrolidone (80 °C, 24 h) under continuous vigorous stirring, yielding a homogeneous solution with a concentration of 16 wt.%. Once all air bubbles were thoroughly eliminated, the solution was poured onto a flat glass plate using a 150 μm-gated casting knife. The glass plate bearing the newly formed film underwent direct drying under vacuum (60 °C, 24 h) and was then immersed in ultrapure water. Subsequently, the dense film was peeled off from the glass plate and underwent multiple rinses with distilled water several times to eliminate any residual NMP solvent. Finally, the PSF films were subjected to an additional 24 h drying period in a vacuum oven at 80 °C to ensure dryness before commencing the surface modification process.

A solution of dopamine (2.0 mg/mL) was freshly prepared by dissolving dopamine in a Tris-buffer solution (50 mM, pH = 8.5). Then, the as-prepared PSF membranes were immersed in the dopamine solution at room temperature in the presence of air. After continuously stirring for 12 h, the membranes were properly cleaned with deionized water to eliminate contaminants and dried overnight at 80 °C under vacuum to obtain polydopamine-modified polysulfone membrane (PSF-PDA).

Surface modification of PSF-PDA was based on the Schiff base reaction. A solution of NH_2_-β-CD with a concentration of 2 mM was obtained in a 50 mM Tris buffer solution (pH = 8.5). The PSF-PDA, prepared as described above, were immersed in this solution with continuously stirring. After reaction for 12 h, the resulting modified membrane was retrieved and subjected to several rinses with deionized water. Finally, cyclodextrin-modified PSF membranes (PSF-CD) were desiccated under vacuum at 80 °C overnight, in preparation for subsequent characterization.

### 2.3. Characterization of Polysulfone Membranes

#### 2.3.1. FT-IR and XPS

ATR–FTIR measurements were carried out on a Nexus 470 FTIR spectrometer (Thermo Nicolet, Detroit, MI, USA) equipped with an ATR cell (ZnSe, 45°). Spectra were acquired over a range of 400 to 4000 cm^−1^, employing 32 scans with a nominal resolution of 4 cm^−1^. XPS spectra were recorded on a PHI-5000C ESCA system (Perkin Elmer, Waltham, MA, USA) with Mg Kα radiation (hν = 1253.6 eV). To mitigate surface charging effects, spectra of all elements were calibrated based on the C 1s hydrocarbon peak at 284.6 eV.

#### 2.3.2. Surface Properties of Polysulfone Membranes

The streaming potential of the membrane surface was monitored using a zeta potential analyzer (Anton Paar, SurPASS, Graz, Austria), utilizing a KCl (1 mmol/L) solution as electrolyte solution. The pH-dependent variation in surface zeta potential was explored by adjusting the pH levels with solutions of NaOH and HCl.

The hydrophilicity of the studied sample was assessed through the measurement of WCA. The WCA was obtained using the sessile drop method and a video-capable CTS-200 contact angle system (MAIST Vision Inspection & Measurement Co., Ltd., Ningbo, China). In an air atmosphere, a 2-μL droplet of ultrapure water was precisely introduced onto a dry sample using a microsyringe. Subsequently, the contact angle (WCA) was computed from the digital image employing DropMeter software (version 2.0). A minimum of five contact angles were averaged to derive a representative value.

#### 2.3.3. BSA Adsorption

Phosphate-buffered saline (PBS) solutions were formulated with the following composition: 1.4 mmol/L KH_2_PO_4_, 2.7 mmol/L KCl, 137 mmol/L NaCl and 4.3 mmol/L Na_2_HPO_4_, and pH was adjusted to 7.4 using 1 M NaOH. Subsequently, BSA solutions with varying concentrations (0.1, 0.2, 0.4, 0.8, 1.2 mg/mL) were freshly prepared and a standard absorbance curve for BSA was established at 280 nm. Typically, after the membrane was pre-washed with PBS buffer, it was submerged in a BSA (1.0 mg/mL) solution and gently agitated at 37 °C for 12 h. Finally, the residual BSA content was determined by assessing the absorption of solution at 280 nm according to the standard curve, and the BSA adsorption properties of different membranes were calculated.

#### 2.3.4. LDL Adsorption

The assessment of LDL adsorption on membrane surface was carried out using ELISA, following the methodology described in our prior research [21,22,23]. The protein solutions relevant need to be prepared freshly prior to each experiment. LDL or HSA solutions are prepared using PBS solution as the base solution. Solutions of the primary antibody was meticulously formulated through the introduction of anti-β-lipoprotein to a PBS solution containing 0.1 wt.% BSA at a dilution ratio of 1:5000. Solutions of the secondary antibody (the peroxidase-conjugated secondary antibody) were obtained by the same method while the dilution ratio was 1:10,000.

The studied samples were cut into 1.4 cm discs and placed in 24-well plates. Following the addition of LDL solutions (500 μL/well) at the designated concentrations, the plate was incubated at 37 °C for 60 min. Following the incubation period, the plate underwent three washes with PBS (1 mL/well). Subsequently, 500 μL of blocking solution (1% BSA in PBS) was added, and the plate was subjected to an additional 30 min incubation at 37 °C. Following an additional three rinses with PBS, the primary antibody (0.5 mL/well) and secondary antibody (0.5 mL/well) were introduced and incubated at 37 °C for 60 min. After each antibody incubation step, a thorough washing procedure was implemented, involving three washes with Tris-buffered saline (4.3 mM Na_2_HPO_4_, 137 mM NaCl, 2.7 mM KCl, 1.4 mM KH_2_PO_4_, 0.1% Tween 20, pH = 7.4). The studied samples were subsequently moved to a blank 24-well plate, and the TMB substrate solution was then added. After 10 min, the chromogenic reaction was stopped with 1 M H_2_SO_4_ solution (0.5 mL/well). Finally, 500 μL of the colored solution was moved to a blank 24-well plate, and the optical density at 450 nm was detected using a microplate reader (ELX800 Reader, BioTek, Winooski, VT, USA).

The experiments of LDL desorption were conducted using a process similar to that employed for the analysis of LDL adsorption with ELISA, as previously elucidated. However, prior to the introduction of primary antibodies, the LDL-incubated membrane underwent a washing step with NaCl or urea solutions, with concentrations varying from 0.25 M to 2 M.

## 3. Results and Discussion

### 3.1. FT-IR/ATR and XPS for Membranes

**Figure 1:**
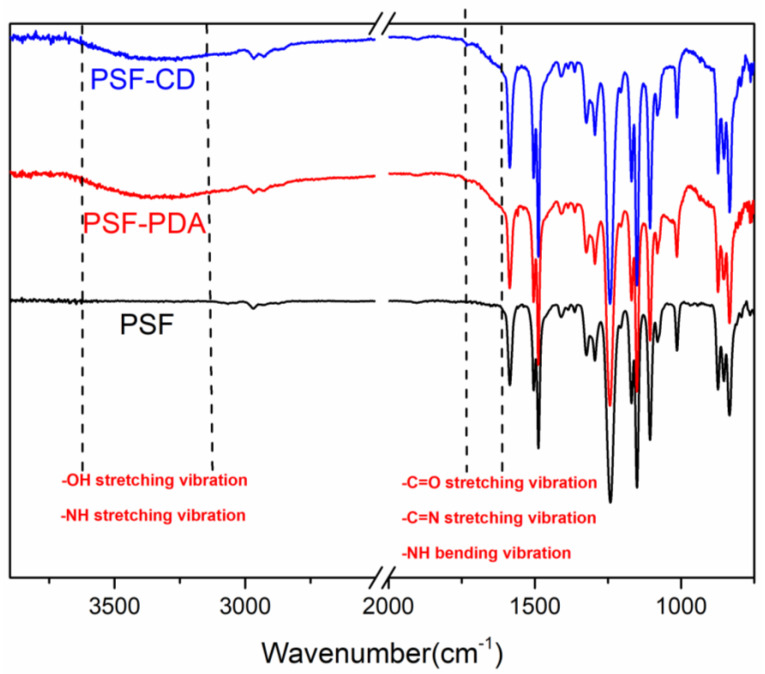
FT-IR/ATR spectra of the PSF, PSF-PDA and PSF-CD.

In this study, chemical changes in the investigated samples were monitored using ATR–FTIR and XPS measurements. As shown in Figure 1, in comparison to the pristine PSF membrane, the PSF-PDA and PSF-CD exhibited a new broad band from 3100 cm^−1^ to 3600 cm^−1^. It should be attributed to the stretching vibration of N-H/O-H. Furthermore, another broad peak signal appeared from 1600 cm^−1^ to 1680 cm^−1^ for PSF-PDA and PSF-CD. This might be an overlap of multiple peaks such as N-H bending vibration, C=O stretching vibration, and C=N stretching vibration, signifying that the PDA layer was generated on the surface of the PSF membrane. The ATR-FTIR spectra of PSF-PDA and PSF-CD showed striking similarities primarily due to their similar chemical composition and the overlapping of characteristic peaks at corresponding positions [48,49].

**Figure 2:**
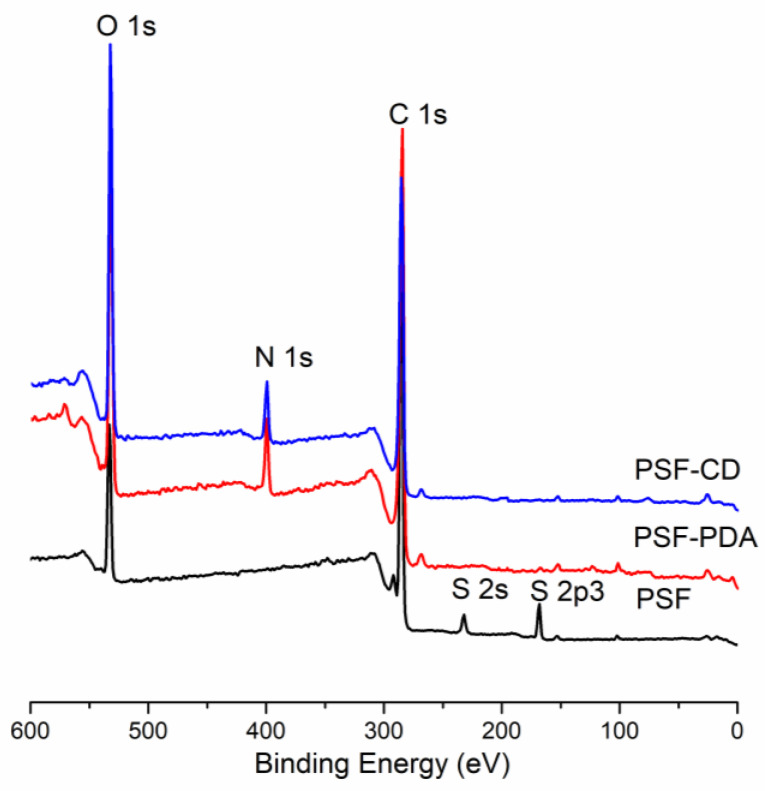
XPS spectra for the surface of black: the blank PSF membrane; red: PDA-modified membrane; blue: cyclodextrin-modified membrane.

The surface elemental composition of the analyzed materials was quantified using XPS measurement (Figure 2), and the corresponding findings obtained from the XPS survey scans are provided in Table 1. As depicted in Figure 2, the PSF-PDA and PSF-CD exhibited a new peak around 401.7 eV, aligning with the binding energy of N1s, when compared to the pristine PSF. The PSF membrane demonstrated elevated levels of C and S (Table 1). After modification using DA, the N content of PSF-PDA increased notably, from 0% to 8.08%, while the content of S decreased significantly from 3.36% to 0.41%, as detailed in Table 1. This result elucidated the successful introduction of a PDA layer onto the surface of the PSF membrane, facilitating further functionalization. After a Schiff base reaction with NH_2_-β-CD, the O content of PSF-CD further increased noticeably from 22.59% to 27.73% and the N content decreased from 8.08% to 7.89%. This phenomenon was primarily attributed to the higher oxygen content and relatively lower nitrogen element content inherent in NH_2_-β-CD, which signified the successful modification of cyclodextrin on the membrane surface. Interestingly, based on the XPS results, the composition of the PSF-CD surface notably deviated from the chemical composition of NH_2_-β-CD, resembling more closely a composite layer of PSF-PDA and PSF-CD. This phenomenon may arise from the X-ray penetration depth surpassing the thickness of the cyclodextrin layer. In addition, the incomplete coverage of the cyclodextrin layer was also a plausible explanation.

To further verify the modification process, high-resolution scans of C 1s, O 1s and N 1s were conducted (Figure S2). The C 1s core level spectrum of PSF exhibited three peaks; the peaks at 284.69 eV and 286.43 eV were assigned to C-C/C-H/C-S and C-O, respectively. The shake-up peak at 291.40 eV was attributed to electron transitions from π to π* of the aromatic ring. The O 1s spectrum of PSF could be deconvoluted into two peaks, identified as C-O-C (peak at 533.55 eV) and S=O (peak at 531.96 eV). No discernible peaks are evident in the N 1s spectrum of PSF due to the absence of nitrogen within the PSF compound.

After modification using DA, a new peak appeared at 288.08 eV in the C 1s spectrum of PSF-PDA, arising from quinone-like structure (C=O) of PDA. In addition, the intensity of the C-O/C-N signal (peak at 286.03 eV) originating from the phenol-like C-OH and the amine moieties was significantly increased. The intensity of the shake-up peak of PSF-PDA was obviously attenuated compared with PSF, indicating the formation of the PDA layer on PSF surface. Accordingly, the S=O signal disappeared and an additional peak assigned to C=O was observed at 531.06 eV in the O 1s spectrum of PSF-PDA. As for the N 1s spectrum of PSF-PDA, three new peaks occurred at 399.91 eV, 401.68 eV and 398.70 eV, respectively. The peaks at 401.68 eV and 399.91 eV were attributed to amino groups such as C-NH-C (cyclic amines) and C-NH_2_ (uncycled amines) and the corresponding protonated amino groups. The binding energy associated with the peak at 398.70 eV exhibited an unusually low value for organically bonded nitrogen species. This anomaly found elucidation in the elevated electron density localized at the nitrogen atom, attributable to the concurrent establishment of an extra π-bond with the adjacent carbon moiety (C–N=C↔C=N–C).

After the Schiff base reaction with NH_2_-β-CD, the shake-up peak completely disappeared in the C 1s spectrum of PSF-CD, indicating the coverage of the cyclodextrin layer on PSF-PDA. This was consistent with the disappearance of C–N=C↔C=N–C peak in the N 1s spectrum of PSF-CD. As an oligosaccharide, cyclodextrin contains numbers of C-O components. Consequently, the intensity of C-O/C-N moiety for PSF-CD (peak at 286.28 eV) noticeably increased further in the C 1s spectrum of PSF-CD compared to PSF-PDA. It was noteworthy that the signal of C=O was still observable in both the C 1s and O 1s core-level spectra of PSF-CD, suggesting that the X-ray penetration depth exceeded the thickness of the cyclodextrin layer. In conclusion, all available results strongly confirmed that cyclodextrins were successfully modified onto the PSF surface.

### 3.2. Surface Properties of the Membranes

**Figure 3:**
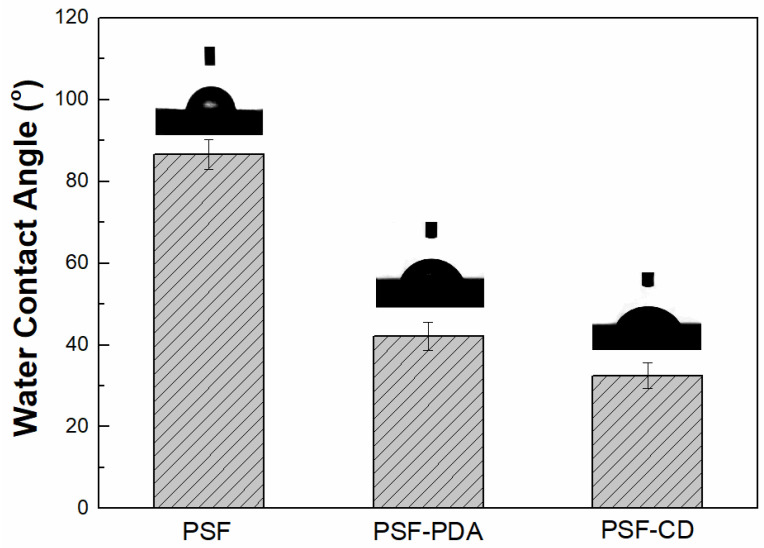
The WCAs of PSF, PSF-PDA and PSF-CD.

The modification of the membrane surface induces shifts in material surface characteristics such as membrane surface charge density, hydrophilicity and so on, which in turn substantiate the efficacy of the modification process. In this paper, the hydrophilicity of the membranes was assessed via WCA measurements. As depicted in Figure 3, it is evident that the membrane’s hydrophilicity undergoes alterations during each modification step. The WCA of the blank polysulfone membrane approached nearly 90°, consistent with findings reported in other studies [47,50]. After modification with dopamine, the WCA of PSF-PDA decreased to 42.1 ± 3.4° as a result of the introduction of multiple hydrophilic groups (such as amino groups or hydroxyl groups) from PDA. Subsequent functionalization of β-cyclodextrin on membrane surface led to a further enhancement of hydrophilicity, evident in the further reduction of the WCA to 32.5 ± 3.2°, ascribed to the further increase in hydrophilic group density.

**Figure 4:**
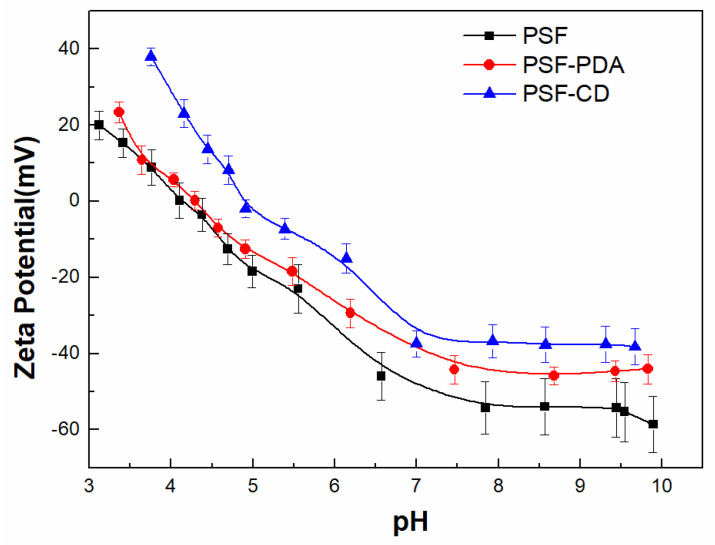
Zeta potential of the PSF, PSF-PDA and PSF-CD under varying pH conditions.

Figure 4 presents the result of the surface potential for studied samples under varying pH conditions (Figure 4). It is found that the Zeta potential of three different PSF membranes showed a similar trend with the pH, and the Zeta potential decreased with the increase in pH. For PSF, the membrane surface is negatively charged in a wide range (pH < 4). Following modification with dopamine, a pronounced elevation in the surface potential was detected for PSF-PDA across the entire pH spectrum. This augmentation in surface potential can be attributed to the existence of positively charged amino groups on the PSF-PDA surface. As for PSF-CD, due to the surface being gradually covered by electrically neutral cyclodextrin molecules, the electronegativity of the membrane surface further decreases. It is noteworthy that all three types of membranes exhibit negative charge surfaces at pH 7.4. Therefore, theoretically, they should exert some electrostatic repulsion toward negatively charged LDL.

### 3.3. BSA Adsorption

**Figure 5:**
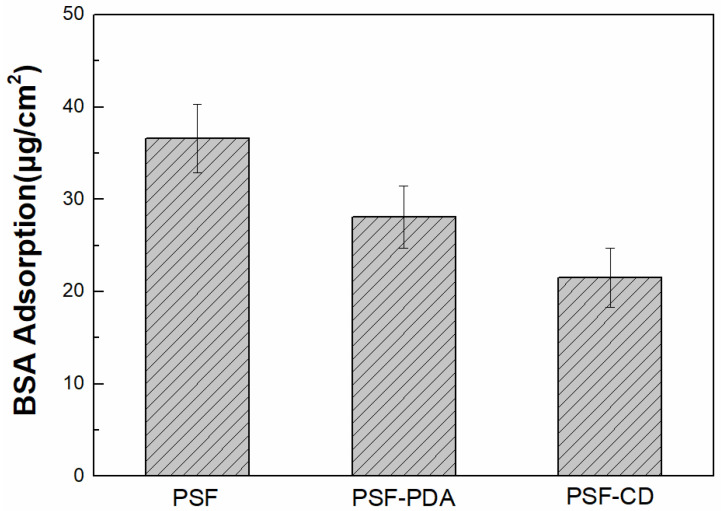
Adsorption of BSA on PSF, PSF-PDA and PSF-CD surfaces.

Biofouling persists as a significant impediment to the dependable performance of biomaterials. Materials exhibiting exceptional properties of anti-specific protein adsorption possess the capacity to effectively avert biofouling and enhance their biocompatibility [51,52]. In this study, Bovine Serum Albumin (BSA) served as a nonspecific protein to scrutinize the adsorption and antifouling characteristics of the modified membranes, as depicted in Figure 5. The quantity of BSA adsorbed onto the membrane was determined using a standard curve (Figure S1), as described in our previous work [50]. Remarkably, the hydrophobic PSF membrane exhibited the highest BSA adsorption when the BSA concentration was maintained at 1 mg/mL. Notably, BSA’s isoelectric point is 4.7, rendering it negatively charged when pH = 7.4. For PSF-PDA and PSF-CD, the surface electronegativity diminishes notably when compared with PSF. Theoretically, this reduction should lead to a decrease in electrostatic repulsion toward BSA. However, owing to the heightened hydrophilicity exhibited by the membrane surface, it demonstrates an enhanced capacity to resist BSA adsorption, resulting in a substantial reduction of BSA adsorption to 28.64 μg/cm^2^ for PSF-PDA and 22.34 μg/cm^2^ for PSF-CD, respectively. Clearly, the ability of anti-non-specific protein adsorption for PSF-CD was significantly improved after modification. Robust resistance to nonspecific protein adsorption can significantly diminish the unspecific binding of other unwanted proteins, thereby markedly enhancing the sensitivity and specificity of membranes for biomedical application. Such attributes are imperative for PSF-CD to selectively adsorb LDL from complex protein solutions.

### 3.4. Adsorption and Desorption of LDL Analysis by ELISA

**Figure 6:**
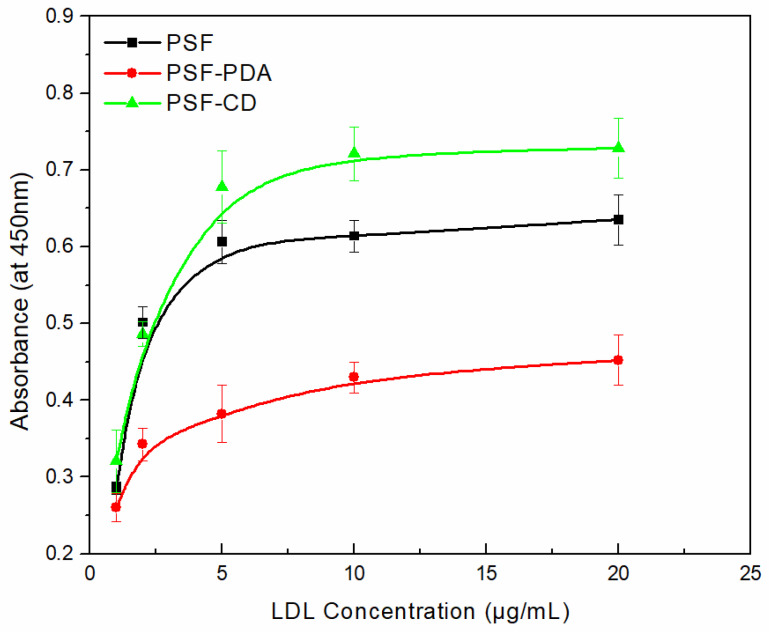
Adsorption of LDL on PSF, PSF-PDA and PSF-CD (LDL = 1, 2, 5, 10, 20 μg/mL).

The enzyme-linked immunosorbent assay, first described by Eva Engvall and Peter Perlmann in 1971, has gained substantial recognition as a biomolecular technique that leverages the specificity of antibodies and the sensitivity of enzyme assays for the detection and quantification of biomolecules [53,54,55,56]. In this study, the amount of LDL adsorbed on the membranes was quantified using optical density measurements obtained through colorimetric ELISA [56]. As depicted in Figure 6, the quantity of LDL adsorption increased in correlation with the concentration of LDL across all samples. This increase underscores the characteristic features of Langmuir-type adsorption with a plateau value, as previously observed in analogous instances of some other protein adsorption onto biomaterials [56,57,58]. Extensive research indicates that protein adsorption on highly hydrophilic surfaces may decrease due to the reduced hydrophobic interactions between the surface and proteins [59,60]. In this study, a comparable phenomenon was witnessed. Compared to PSF, the absorbance noticeably decreased for PSF-PDA after exposure to the same LDL solution, signifying reduced LDL adsorption. This phenomenon can be attributed to enhanced hydrophilicity, as evidenced by WCA measurements. However, such is not the case for the PSF-CD. It is evident that the PSF-CD exhibited a higher LDL adsorption capacity compared to the PSF and the PSF-PDA. This phenomenon should be ascribed to the hydrophobic cavity of cyclodextrin, which exerts a strong hydrophobic force with lipids situated on the LDL surface. Additionally, the abundance of hydroxyl groups on the outer edge of cyclodextrin facilitates hydrogen bonding with phospholipids located on the LDL surface.

The composition of the adsorbed protein layer on biomaterial surface is influenced by multiple interactions such as electrostatic interaction, hydrogen bonding, hydrophobic interaction and so on. In pursuit of a deeper understanding of the predominant interaction force existing between the LDL and the surface, various concentrations of NaCl or urea solutions were employed as eluents for desorption experiments on different polysulfone membranes. The introduction of NaCl serves to modulate the ionic strength of the solution, thereby affecting the electrostatic interactions between the membrane and LDL. Urea, known for its efficacy in disrupting hydrogen bonds, exerts an influence on the hydrogen bonding interactions between the surface and the LDL.

**Figure 7:**
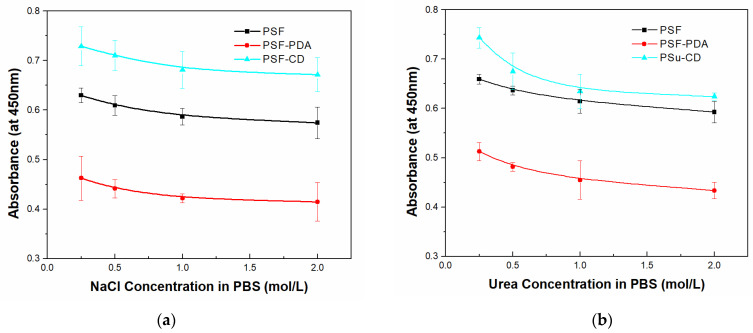
Single protein adsorption (LDL = 10 μg/mL) and then washed with NaCl (**a**) or urea (**b**) solutions of various concentrations on different modified PSF membranes.

Figure 7a elucidates the result of desorption of LDL rinsed with NaCl solutions. All three samples exhibit partial desorption of adsorbed LDL when exposed to NaCl solutions, indicating that the LDL previously adsorbed on the membrane surface was partly affected by electrostatic interaction. Despite the fact that all three samples display a negatively charged surface, as revealed by zeta potential measurements, they still lack negatively ionizable groups to induce strong electrostatic interaction. Therefore, all the LDL desorption curves showed relatively minor changes with increasing NaCl concentration. Figure 7b shows the results of LDL desorption using urea as an eluent. As can be seen, the elution curve of PSF-CD is more significantly influenced by urea compared with PSF and PSF-PDA. Mascetti et al. emphasized that, as phospholipids form complexes within the cavity of cyclodextrin, the hydrogen bonding interaction between the negatively charged phosphate groups on their surface and the hydroxyl groups at the outer edge of cyclodextrin also contribute to the stability of these complexes [61]. Hence, the urea-induced LDL desorption primarily results from urea’s interference with the hydrogen bonding interactions between the surface hydroxyl groups of PSF-CD and phospholipids, as well as other components on the LDL surface.

The adsorption of proteins onto surfaces of biomaterials in vivo is commonly concomitant with protein competition. Nonetheless, the replication of such competitive processes using complex protein mixtures poses a formidable challenge in terms of controlling all the essential variables. Consequently, adsorption studies employing mixtures of two proteins are commonly applied to streamline the evaluation. In this study, HAS was chosen as the competitor due to its abundant presence in human blood. Mixed solutions of LDL and HAS were freshly prepared and the films were immersed in the solutions. In accordance with the Vroman effect, the composition of the adsorbed protein layer varies with the duration of interaction. Proteins with elevated concentrations or greater mobility initially predominated the surface, owing to a heightened likelihood of collisions or more rapid diffusion rates. However, they were subsequently displaced by less mobile proteins exhibiting a higher affinity for the surface.

**Figure 8:**
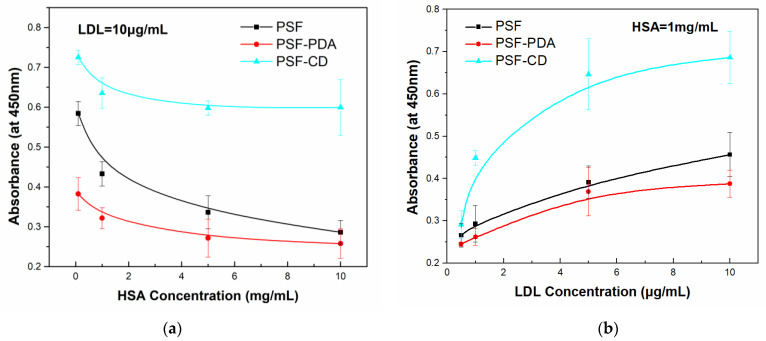
Adsorption of LDL on different PSF membranes from binary-protein solutions of LDL and HAS: (**a**) fixed LDL concentration, varying HSA concentration, (**b**) fixed HSA concentration, varying LDL concentration.

Figure 8 shows the results of competitive adsorption originating from binary solutions onto membrane surfaces. When the concentration of LDL remained constant at 10 μg/mL while the HSA concentration varied, the introduction of HSA significantly attenuated the adsorption of LDL to the PSF, as can be seen in Figure 8a. However, the interference of HSA seems to have a lesser effect on the adsorption of LDL to PSD-CD. The PSF-CD films exhibited a pronounced affinity for LDL, even when exposed to high concentrations of HSA at 10 mg/mL. Conversely, the PSF and PSF-PDA films displayed minimal adsorption under identical conditions. It is important to highlight that this corresponds to a HSA to LDL ratio of 1000:1, a significantly higher ratio compared to that typically observed in the plasma of healthy humans (the levels of HSA and LDL were 30–50 g/L and 100–120 mg/mL [53,54], respectively). This result signifies that PSF-CD maintains a remarkable adsorption capability for LDL, even in the presence of a substantial interference from a high concentration of HAS.

To further investigate the impact of an elevated LDL to HSA ratio on LDL binding, the concentration of HSA was held constant at 1.0 mg/mL and the concentration of LDL was changed from 0 to 10 μg/mL, as illustrated in in Figure 8b. It was observed that PSF and PSF-PDA exhibited minimal increases in LDL adsorption compared to PSF-CD, which displayed a noticeable increase in adsorption with rising LDL concentration. An evident increase in the binding of LDL was noted as LDL concentration progressively increased, reaching a ratio of LDL to HSA at 1:100. As previously mentioned, the ratio of LDL to HSA in plasma typically falls within the range of 1:30 to 1:50 for individuals in good health. However, this ratio significantly elevates in hypercholesterolemic patients, exceeding 160 mg/dL in LDL level. The ability of PSF-CD to selectively adsorb LDL from binary-protein solutions indicates that this material has the potential to be highly effective for LDL adsorption in pathological conditions.

## 4. Conclusions

In this study, we proposed a facile and efficient approach for immobilizing cyclodextrin onto PSF membranes for selective adsorption of LDL, which involved the modification of PDA via dopamine self-assembly and the functionalization of cyclodextrin through a Schiff base reaction with NH_2_-β-CD. The successful implementation of the two-step modification process was validated through the results of ATR-FTIR, XPS and Zeta potential measurements. The hydrophilicity of the PSF-CD was significantly enhanced, as evidenced by a notable reduction in the water contact angle from 86.6 ± 3.7° (PSF) to 32.5 ± 3.2° (PSF-CD) following the binding of cyclodextrin to PSF membranes.

The results of ELISA demonstrated that the obtained PSF-CD showed excellent adsorption capacity for LDL. In contrast to conventional negatively charged LDL adsorbents, electrostatic forces do not play a dominant role. Instead, hydrogen bonding has a more significant impact on the adsorption of LDL to the material surface. The PSF-CD possessed remarkable adsorption capacity and higher affinity for LDL in both single-protein and binary-protein solutions. Therefore, it can be concluded that PSF-CD holds substantial potential for application in simultaneous hemodialysis and LDL apheresis therapy.

## Figures and Tables

**Table 1:** Surface composition of the blank, dopamine-modified and cyclodextrin-modified PSF membranes.

Samples	Atomic (wt.%)			
	C	O	N	S
PSF	81.58%	15.06%	-	3.36%
PSF-PDA	68.92%	22.59%	8.08%	0.41%
PSF-CD	64.16%	27.73%	7.89%	0.22%

## Data Availability

Data are contained within the article.

## Supplementary Materials

The following supporting information can be downloaded at: https://www.mdpi.com/article/10.3390/ma17050988/s1, Figure S1. Standard curves of the BSA adsorption. Figure S2. High-resolution XPS core-level spectra of C 1s, O 1s, N 1s for original, dopamine-modified and cyclodextrin-modified 21 PSF membranes.

## References

[1] Paukner K., Králová Lesná I., Poledne R. (2022). Cholesterol in the Cell Membrane—An Emerging Player in Atherogenesis. Int. J. Mol. Sci..

[2] Murtola T., Vuorela T.A., Hyvönen M.T., Marrink S.-J., Karttunen M., Vattulainen I. (2011). Low Density Lipoprotein: Structure, Dynamics, and Interactions of apoB-100 with Lipids. Soft Matter.

[3] Prassl R., Laggner P., Prassl R., Laggner P. (2012). Lipoprotein Structure and Dynamics: Low Density Lipoprotein Viewed as a Highly Dynamic and Flexible Nanoparticle. Lipoproteins—Role in Health and Diseases.

[4] Kiberstis P.A. (2019). Fatty Liver—Too Much of a Bad Thing?. Science.

[5] Thierer J.H., Ekker S.C., Farber S.A. (2019). The LipoGlo Reporter System for Sensitive and Specific Monitoring of Atherogenic Lipoproteins. Nat. Commun..

[6] Tolfrey K., Jones A.M., Campbell I.G. (2000). The Effect of Aerobic Exercise Training on the Lipid-Lipoprotein Profile of Children and Adolescents. Sports Med..

[7] Gylling H., Plat J., Turley S., Ginsberg H.N., Ellegård L., Jessup W., Jones P.J., Lütjohann D., Maerz W., Masana L. (2014). Plant Sterols and Plant Stanols in the Management of Dyslipidaemia and Prevention of Cardiovascular Disease. Atherosclerosis.

[8] Nicolosi R.J., Stucchi A.F., Kowala M.C., Hennessy L.K., Hegsted D.M., Schaefer E.J. (1990). Effect of Dietary Fat Saturation and Cholesterol on LDL Composition and Metabolism. In Vivo Studies of Receptor and Nonreceptor-Mediated Catabolism of LDL in Cebus Monkeys. Arterioscler. Off. J. Am. Heart Assoc. Inc..

[9] Tsouli S.G., Xydis V., Argyropoulou M.I., Tselepis A.D., Elisaf M., Kiortsis D.N. (2009). Regression of Achilles Tendon Thickness after Statin Treatment in Patients with Familial Hypercholesterolemia: An Ultrasonographic Study. Atherosclerosis.

[10] Samuel E., Watford M., Egolum U.O., Ombengi D.N., Ling H., Cates D.W. (2023). Inclisiran: A First-in-Class siRNA Therapy for Lowering Low-Density Lipoprotein Cholesterol. Ann. Pharmacother..

[11] Ballantyne C.M., Bays H.E., Louie M.J., Smart J., Zhang Y., Ray K.K. (2022). Factors Associated with Enhanced Low-Density Lipoprotein Cholesterol Lowering With Bempedoic Acid. J. Am. Heart Assoc..

[12] Huddy K., Dhesi P., Thompson P.D. (2013). Do the Frequencies of Adverse Events Increase, Decrease, or Stay the Same with Long-Term Use of Statins?. Curr. Atheroscler. Rep..

[13] Kasteleyn M.J., Wezendonk A., Vos R.C., Numans M.E., Jansen H., Rutten G.E. (2014). Repeat Prescriptions of Guideline-Based Secondary Prevention Medication in Patients with Type 2 Diabetes and Previous Myocardial Infarction in Dutch Primary Care. Fam. Pract..

[14] van Wijk D.F., Sjouke B., Figueroa A., Emami H., van der Valk F.M., MacNabb M.H., Hemphill L.C., Schulte D.M., Koopman M.G., Lobatto M.E. (2014). Nonpharmacological Lipoprotein Apheresis Reduces Arterial Inflammation in Familial Hypercholesterolemia. J. Am. Coll. Cardiol..

[15] Makino H., Koezuka R., Tamanaha T., Ogura M., Matsuki K., Hosoda K., Harada-Shiba M. (2019). Familial Hypercholesterolemia and Lipoprotein Apheresis. J. Atheroscler. Thromb..

[16] Winters J.L. (2011). Lipid Apheresis, Indications, and Principles. J. Clin. Apheresis.

[17] Julius U., Fischer S., Schatz U., Hohenstein B., Bornstein S.R. (2013). Lipoprotein Apheresis: An Update. Clin. Lipidol..

[18] Taylan C., Weber L.T. (2023). An Update on Lipid Apheresis for Familial Hypercholesterolemia. Pediatr. Nephrol..

[19] Richter W.O., Jacob B.G., Ritter M.M., Sühler K., Vierneisel K., Schwandt P. (1993). Three-Year Treatment of Familial Heterozygous Hypercholesterolemia by Extracorporeal Low-Density Lipoprotein Immunoadsorption with Polyclonal Apolipoprotein B Antibodies. Metabolism.

[20] Bereli N., Şener G., Yavuz H., Denizli A. (2011). Oriented Immobilized Anti-LDL Antibody Carrying Poly (Hydroxyethyl Methacrylate) Cryogel for Cholesterol Removal from Human Plasma. Mater. Sci. Eng. C.

[21] Huang X.-J., Guduru D., Xu Z.-K., Vienken J., Groth T. (2010). Immobilization of Heparin on Polysulfone Surface for Selective Adsorption of Low-Density Lipoprotein (LDL). Acta Biomater..

[22] Huang X.-J., Guduru D., Xu Z.-K., Vienken J., Groth T. (2011). Blood Compatibility and Permeability of Heparin-Modified Polysulfone as Potential Membrane for Simultaneous Hemodialysis and LDL Removal. Macromol. Biosci..

[23] Li J., Huang X.-J., Ji J., Lan P., Vienken J., Groth T., Xu Z.-K. (2011). Covalent Heparin Modification of a Polysulfone Flat Sheet Membrane for Selective Removal of Low-Density Lipoproteins: A Simple and Versatile Method. Macromol. Biosci..

[24] Hou X., Zhang T., Cao A. (2013). A Heparin Modified Polypropylene Non-Woven Fabric Membrane Adsorbent for Selective Removal of Low Density Lipoprotein from Plasma. Polym. Adv. Technol..

[25] Cao J.-F., Xu W., Zhang Y.-Y., Shu Y., Wang J.-H. (2021). Chondroitin Sulfate-Enriched Hierarchical Multichannel Polydopamine Nanoparticles with Ultrahigh Sorption Capacity for Separation of Low-Density Lipoprotein. J. Mater. Chem. B.

[26] Cao J.-F., Xu W., Zhang Y.-Y., Shu Y., Wang J.-H. (2021). A Salt Stimulus-Responsive Nanohydrogel for Controlled Fishing Low-Density Lipoprotein with Superior Adsorption Capacity. ACS Appl. Mater. Interfaces.

[27] Othi N.A., Hanan A., Solangi M.Y., AlSalhi M.S., Devanesan S., Shar M.A., Bhutto M.A., Abro M.I., Aftab U. (2023). Facile Preparation of Amino Acid-Assisted Fe_3_O_4_ Nanoparticles for Low-Density Lipoprotein Cholesterol Removal. Chem. Pap..

[28] Yu Y., Dong J., Ma B., Jiang X., Guo C., Liu Z., Chai Y., Wang L., Sun L., Ou L. (2021). Bio-Inspired Dual-Functional Phospholipid–Poly(Acrylic Acid) Brushes Grafted Porous Poly(Vinyl Alcohol) Beads for Selective Adsorption of Low-Density Lipoprotein. J. Mater. Chem. B.

[29] Yu Y., Ma B., Guo C., Jiang X., Liu Z., Chai Y., Wang L., Du Y., Wang B., Li N. (2022). Biomembrane-mimetic Hemoperfusion Adsorbent for Efficient Removal of Low-density Lipoprotein from Hyperlipemia Blood. J. Biomed. Mater. Res. B Appl. Biomater..

[30] Fu G., Li H., Yu H., Liu L., Yuan Z., He B. (2006). Synthesis and Lipoprotein Sorption Properties of Porous Chitosan Beads Grafted with Poly(Acrylic Acid). React. Funct. Polym..

[31] Xu Y., Li Y., Zhao W., Zhao C. (2023). Simple Emulsion Template Method towards Self-Anticoagulant and High-Efficiency Carboxymethyl Chitosan-Based Adsorbent for Low-Density Lipoprotein from Whole Blood. J. Colloid Interface Sci..

[32] Li J., Huang X.-J., Vienken J., Xu Z.-K., Groth T. (2013). Bioinspired Multiple-Interaction Model Revealed in Adsorption of Low-Density Lipoprotein to Surface Containing Saccharide and Alkanesulfonate. Langmuir.

[33] Fang F., Zhu X.-Y., Chen C., Li J., Chen D.-J., Huang X.-J. (2017). Anionic Glycosylated Polysulfone Membranes for the Affinity Adsorption of Low-Density Lipoprotein via Click Reactions. Acta Biomater..

[34] Liu Z., Ye L., Xi J., Wang J., Feng Z. (2021). Cyclodextrin Polymers: Structure, Synthesis, and Use as Drug Carriers. Prog. Polym. Sci..

[35] Cid-Samamed A., Rakmai J., Mejuto J.C., Simal-Gandara J., Astray G. (2022). Cyclodextrins Inclusion Complex: Preparation Methods, Analytical Techniques and Food Industry Applications. Food Chem..

[36] Mejia-Ariza R., Graña-Suárez L., Verboom W., Huskens J. (2017). Cyclodextrin-Based Supramolecular Nanoparticles for Biomedical Applications. J. Mater. Chem. B.

[37] Alsbaiee A., Smith B.J., Xiao L., Ling Y., Helbling D.E., Dichtel W.R. (2016). Rapid Removal of Organic Micropollutants from Water by a Porous β-Cyclodextrin Polymer. Nature.

[38] Szejtli J. (1998). ChemInform Abstract: Introduction and General Overview of Cyclodextrin Chemistry. ChemInform.

[39] Sinha A., Basiruddin S., Chakraborty A., Jana N.R. (2015). β-Cyclodextrin Functionalized Magnetic Mesoporous Silica Colloid for Cholesterol Separation. ACS Appl. Mater. Interfaces.

[40] Huang Z., London E. (2013). Effect of Cyclodextrin and Membrane Lipid Structure upon Cyclodextrin–Lipid Interaction. Langmuir.

[41] Weitzer A., Katsaras J., Heberle F.A. (2018). Cyclodextrin-Mediated Lipid Exchange Monitored with FRET. Biophys. J..

[42] Szente L., Fenyvesi É. (2017). Cyclodextrin-Lipid Complexes: Cavity Size Matters. Struct. Chem..

[43] Hoang Thi T.T., Lee J.S., Lee Y., Park H., Park K., Park K. (2017). Supramolecular Cyclodextrin Supplements to Improve the Tissue Adhesion Strength of Gelatin Bioglues. ACS Macro Lett..

[44] Fang F., Huang X.-J., Guo Y.-Z., Hong X., Wu H.-M., Liu R., Chen D.-J. (2018). Selective and Regenerable Surface Based on β-Cyclodextrin for Low-Density Lipoprotein Adsorption. Langmuir.

[45] Wu H., Fang F., Wang C., Hong X., Chen D., Huang X. (2021). Selective Molecular Recognition of Low Density Lipoprotein Based on β-Cyclodextrin Coated Electrochemical Biosensor. Biosensors.

[46] Bakris G.L., Ritz E. (2009). The Message for World Kidney Day 2009: Hypertension and Kidney Disease—A Marriage That Should Be Prevented. J. Hum. Hypertens..

[47] Wang L., Fang F., Liu Y., Li J., Huang X. (2016). Facile Preparation of Heparinized Polysulfone Membrane Assisted by Polydopamine/Polyethyleneimine Co-Deposition for Simultaneous LDL Selectivity and Biocompatibility. Appl. Surf. Sci..

[48] Fang J., Chen Y., Fang C., Zhu L. (2022). Regenerable Adsorptive Membranes Prepared by Mussel-Inspired Co-Deposition for Aqueous Dye Removal. Sep. Purif. Technol..

[49] Wu H., Kong J., Yao X., Zhao C., Dong Y., Lu X. (2015). Polydopamine-Assisted Attachment of β-Cyclodextrin on Porous Electrospun Fibers for Water Purification under Highly Basic Condition. Chem. Eng. J..

[50] Fang F., Zhao H.-Y., Wang R., Chen Q., Wang Q.-Y., Zhang Q.-H. (2023). Fabrication and Study of Dextran/Sulfonated Polysulfone Blend Membranes for Low-Density Lipoprotein Adsorption. Materials.

[51] Gnanasampanthan T., Karthäuser J.F., Spöllmann S., Wanka R., Becker H.-W., Rosenhahn A. (2022). Amphiphilic Alginate-Based Layer-by-Layer Coatings Exhibiting Resistance against Nonspecific Protein Adsorption and Marine Biofouling. ACS Appl. Mater. Interfaces.

[52] Kruk T., Bzowska M., Hinz A., Szuwarzyński M., Szczepanowicz K. (2021). Control of Specific/Nonspecific Protein Adsorption: Functionalization of Polyelectrolyte Multilayer Films as a Potential Coating for Biosensors. Materials.

[53] Wild C.P., Jiang Y.-Z., Sabbioni G., Chapot B., Montesano R. (1990). Evaluation of Methods for Quantitation of Aflatoxin-Albumin Adducts and Their Application to Human Exposure Assessment. Cancer Res..

[54] Makogonenko E., Tsurupa G., Ingham K., Medved L. (2002). Interaction of Fibrin (Ogen) with Fibronectin: Further Characterization and Localization of the Fibronectin-Binding Site. Biochemistry.

[55] MacMullan M.A., Ibrayeva A., Trettner K., Deming L., Das S., Tran F., Moreno J.R., Casian J.G., Chellamuthu P., Kraft J. (2020). ELISA Detection of SARS-CoV-2 Antibodies in Saliva. Sci. Rep..

[56] Wang W., Huang X.-J., Cao J.-D., Lan P., Wu W. (2014). Immobilization of Sodium Alginate Sulfates on Polysulfone Ultrafiltration Membranes for Selective Adsorption of Low-Density Lipoprotein. Acta Biomater..

[57] Nakamura K., Matsumoto K. (2006). Protein Adsorption Properties on a Microfiltration Membrane: A Comparison between Static and Dynamic Adsorption Methods. J. Membr. Sci..

[58] Saraydin D., Karadaǧ E., Öztop H.N., Güven O. (1994). Adsorption of Bovine Serum Albumin onto Acrylamid—Maleic Acid Hydrogels. Biomaterials.

[59] Bayramoğlu G., Yalçin E., Arica M.Y. (2005). Adsorption of Serum Albumin and γ-Globulin from Single and Binary Mixture and Characterization of pHEMA-Based Affinity Membrane Surface by Contact Angle Measurements. Biochem. Eng. J..

[60] Higuchi A., Hashiba H., Hayashi R., Yoon B.O., Sakurai M., Hara M. (2004). Serum Protein Adsorption and Platelet Adhesion on Aspartic-Acid-Immobilized Polysulfone Membranes. J. Biomater. Sci. Polym. Ed..

[61] Mascetti J., Castano S., Cavagnat D., Desbat B. (2008). Organization of β-Cyclodextrin under Pure Cholesterol, DMPC, or DMPG and Mixed Cholesterol/Phospholipid Monolayers. Langmuir.

