# Supplementary material for: Facile Preparation of β-Cyclodextrin-Modified Polysulfone Membrane for Low-Density Lipoprotein Adsorption via Dopamine Self-Assembly and Schiff Base Reaction
Source: Materials (Basel). 2024 Feb 21;17(5):988. doi: 10.3390/ma17050988 (PMC10934633; doi:10.3390/ma17050988)
Supplement: Supplementary file 1 [file materials-17-00988-s001.zip › materials-2732992-supplementary.pdf]

materials

MDPI

Supplementary Materials

Facile Preparation of β-Cyclodextrin-Modified Polysulfone Membrane for Low-Density Lipoprotein Adsorption via Dopamine Self-Assembly and Schiff Base Reaction

Fei Fang [1,2], Haiyang Zhao [2], Rui Wang [2], Qi Chen [2], Qiongyan Wang [2,*] and Qinghua Zhang [1,*]

[1] College of Chemical and Biological Engineering, Zhejiang University, Hangzhou 310027, China; 3090102659@zju.edu.cn

[2] Research and Development Center, Zhejiang Sucon Silicone Co., Ltd., Shaoxing 312088, China; hycsea@163.com (H.Z.); 13989503732@163.com (R.W.); 13989552135@163.com (Q.C.)

* Correspondence: wqy2040@163.com (Q.W.); qhzhang@zju.edu.cn (Q.Z.)

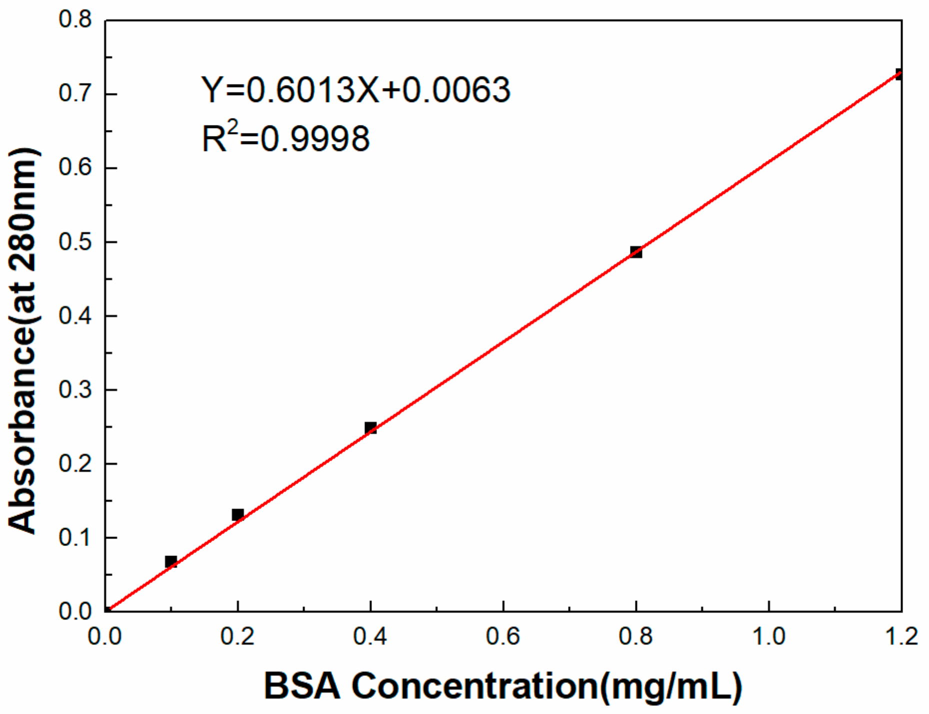

Figure S1. Standard curves of the BSA adsorption.

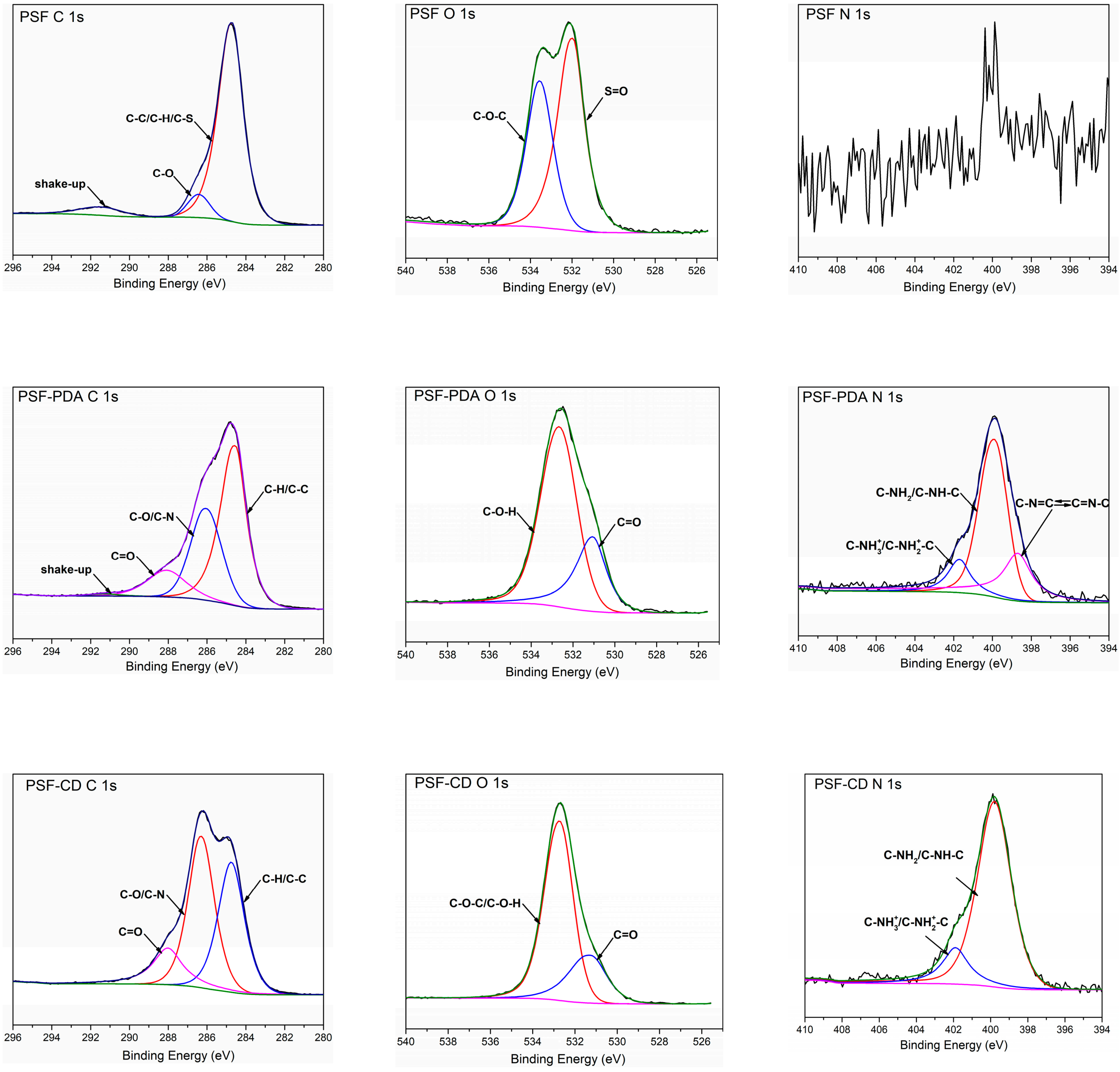

Figure S2. High-resolution XPS core-level spectra of C 1s, O 1s, N 1s for original, dopamine-modified and cyclodextrin-modified PSF membranes.
